# Speciation patterns and processes in the zooplankton of the ancient lakes of Sulawesi Island, Indonesia

**DOI:** 10.1002/ece3.697

**Published:** 2013-08-01

**Authors:** James J Vaillant, Dan G Bock, G Douglas Haffner, Melania E Cristescu

**Affiliations:** 1 Great Lakes Institute for Environmental Research, University of WindsorWindsor, Ontario, N9B 3P4, Canada; 2 Department of Botany, University of British ColumbiaVancouver, British Columbia, V6T 1Z4, Canada; 3 Department of Biology, McGill UniversityMontreal, Quebec, H3A 1B1, Canada

**Keywords:** Ancient lakes, copepoda, Malili lakes, plankton, population structure

## Abstract

Although studies of ancient lake fauna have provided important insights about speciation patterns and processes of organisms in heterogeneous benthic environments, evolutionary forces responsible for speciation in the relatively homogenous planktonic environment remain largely unexplored. In this study, we investigate possible mechanisms of speciation in zooplankton using the freshwater diaptomids of the ancient lakes of Sulawesi, Indonesia, as a model system. We integrate phylogenetic and population genetic analyses of mitochondrial and nuclear genes with morphological and genome size data. Overall, our results support the conclusion that colonization order and local adaptation are dominant at the large, island scale, whereas at local and intralacustrine scales, speciation processes are regulated by gene flow among genetically differentiated and locally adapted populations. In the Malili lakes, the diaptomid populations are homogenous at nuclear loci, but show two highly divergent mitochondrial clades that are geographically restricted to single lakes despite the interconnectivity of the lake systems. Our study, based on coalescent simulations and population genetic analyses, indicates that unidirectional hybridization allows gene flow across the nuclear genome, but prevents the introgression of mitochondria into downstream populations. We suggest that hybridization and introgression between young lineages is a significant evolutionary force in freshwater plankton.

## Introduction

A fundamental goal of evolutionary biology is to understand the forces that develop species and maintain biodiversity. For hundreds of years, evolutionists have studied species radiations across isolated island archipelagos to investigate and test predictions about speciation processes. Similarly, ancient, long-lived lakes (>500,000 years old) represent the aquatic equivalents of islands and have become the focus of many current empirical speciation researchers. In these unique habitats, species are often found to rapidly radiate ecologically and form many flocks of closely related lineages (Martens 1997; Cristescu et al. 2010) making them ideal systems for comparative study and testing theoretical predictions about speciation. Important insights about the roles of habitat isolation, disruptive natural and sexual selection, and hybridization have helped to develop specific models of speciation. Extrinsic forces like lake-level fluctuations in response to climatic shifts (Johnson et al. 1996; Abbott et al. 1997; Cohen et al. 1997; Dumont 1998; Scholz et al. 2007) have influenced the evolution of lacustrine fauna through periods of allopatry, population bottlenecks, and alternate selection regimes (e.g., Rüber et al. 1999; Cristescu et al. 2003; Genner et al. 2010). Recent studies in ancient lakes revealed examples of sympatric speciation in which natural and/or sexual selection play a dominant role in shaping diversification in the face of significant initial gene flow (e.g., Schliewen and Klee 2004; Herder and Schliewen 2010). Moreover, introgressive hybridization between closely related lineages of ancient lakes is now recognized as a significant force of diversification by introducing greater phenotypic diversity (Salzburger et al. 2002; Smith et al. 2003; Seehausen 2004; Bell and Travis 2005; Herder et al. 2006; Koblmüller et al. 2007; Stelkens et al. 2009; Joyce et al. 2011).

Most ancient lakes have simple food webs and planktonic communities consisting of a single endemic grazing calanoid and small number of predatory species (Dumont 1994; Doi et al. 2012). Dumont (1994) hypothesized that simplified zooplankton communities are the result of competitive exclusion. He reasoned that competition is sharpest in long-lived (time not limiting), tropical (low seasonal variation), deep (narrow resource base) lakes, so that an “equilibrium” state can nearly be reached. These observations led to the hypothesis that the homogeneity of the pelagic habitat limits niche diversification. Nevertheless, radiations characterized by striking morphological divergence over very short timescales (i.e., since the Pleistocene) and relatively low molecular divergences have occurred in pelagic species of the Ponto-Caspian region, including mysids, cyclopoids, and onychopods (Väinölä 1995; Monchenko 1998; Cristescu and Hebert 2002, 2005). However, the various physical and ecological forces that drive speciation (e.g., genetic drift, gene flow among differentiated lineages, natural and sexual selection) in pelagic organisms remain unclear.

**Figure 1:**
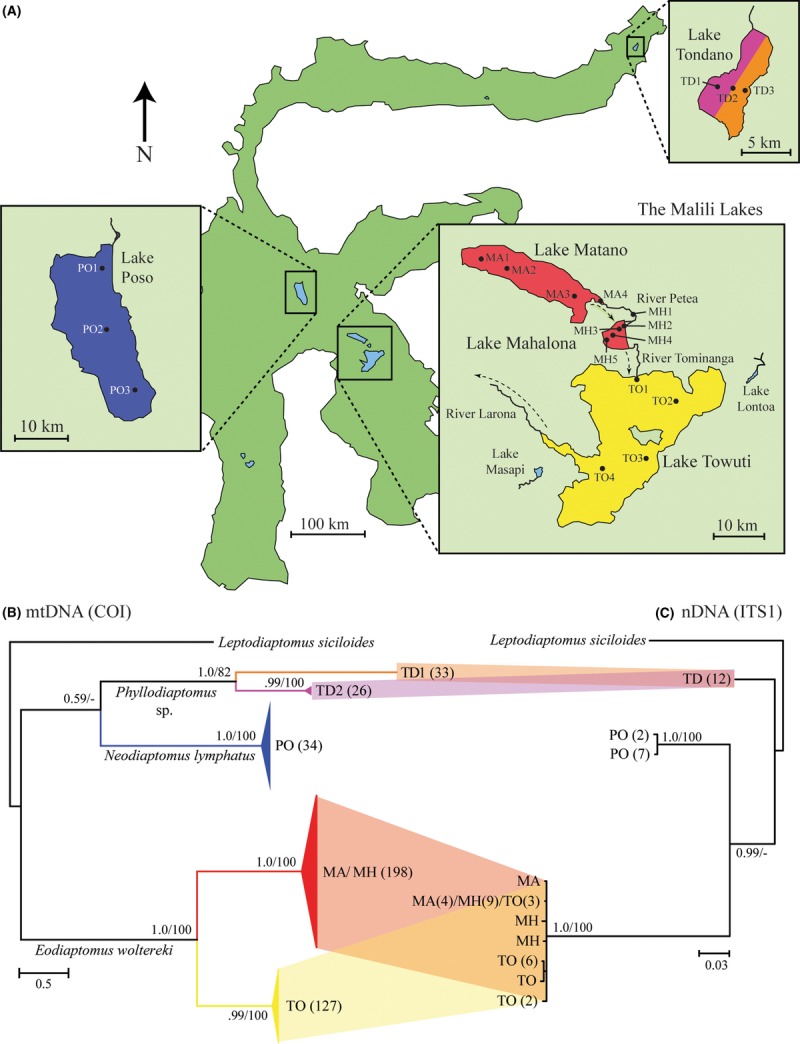
(A) A map of sampling sites across Sulawesi, Indonesia. The dashed arrows indicate the direction of water flow. The Bayesian (BI) phylogeny for the cytochrome *c* oxidase subunit I (COI) gene (B) shows two divergent mitochondrial lineages inhabiting Lake Tondano and two in the Malili lakes. These lineages collapse in the BI phylogeny for the ITS1 region (C) suggesting that hybridization has homogenized the ribosomal genes. Numbers in parentheses indicate the number of sampled individuals within the clade or haplotype. Node supports are BI posterior probabilities followed by neighbor-joining bootstrap values.

The large lakes of Sulawesi centered in the unique biogeographic region of Wallacea have peculiar ecological characteristics. In this study we compare three isolated lake systems (i.e., Tondano, Poso, and Malili; Fig. 1A) which are located in separate watersheds that have different geological histories (Moss and Wilson 1998). Although tropical lakes typically have high primary productivity (Lewis 1996), Lake Poso and the Malili lakes are ultraoligotrophic and have extremely low phytoplankton biomass (Lehmusluoto 1997; Haffner et al. 2001; Sabo et al. 2008) and simple food webs. Much like the rest of Sulawesi, the biological assemblages of these lakes are characterized by a very high degree of endemism (Whitten et al. 1987). These unique environmental and ecological characteristics are the result of the tectonic origin of the lakes combined with the biogeographic processes of Wallacea (Whitten et al. 1987; Lehmusluoto 1997). By contrast, Lake Tondano is eutrophic and has been repeatedly affected by volcanism in the region (Lehmusluoto 1997; Dam et al. 2001).

The ancient Malili lakes (Matano, Mahalona, and Towuti; Fig. 1A) are the only hydrologically interconnected ancient lakes on Earth (Brooks 1950). The Malili catchment basin is composed of ultrabasic and calcareous rock that is rich in heavy metals (Haffner et al. 2001) including calcium, strontium, barium, chromium, and iron which differ as much as twofold in concentration among the lakes (G. D. Haffner, unpubl. data). About one-third of the Malili lakes' diatom species are endemic to single lakes, further indicating that the physicochemical environments vary in ways that limit the success of different taxa among lakes (Bramburger et al. 2008). Lake Matano is a steep-sided graben lake estimated around 1–4 million years old that extends to a depth of 590 m (Haffner et al. 2001). The lake is meromictic with a persistent pycnocline between 100 and 250 m and features some of the highest iron concentrations of any freshwater lake (Crowe et al. 2008), potentially toxic concentrations of chromium, and limiting levels of phosphorus and nitrogen (Sabo et al. 2008). By contrast, lakes Mahalona and Towuti are polymictic and well oxygenated throughout their 60 and 200 m depths, respectively (Sabo 2006). Lake Towuti is likely <1 million years old based on sedimentary characteristics and supports a larger forage fish community than Lake Matano, as evidenced by the presence of active fisheries (Parenti and Soeroto 2004; Walter et al. 2011).

The Malili lakes have been the site of many adaptive radiations including telmatherinid fishes, gastropods, shrimps, and crabs (reviewed in Vaillant et al. 2011; von Rintelen et al. 2011). Striking cases of adaptive evolution associated with feeding morphology or habitat preference have been found in the shrimp, gastropod, crab, and fish species of the lakes (Glaubrecht and von Rintelen 2008; Schubart and Ng 2008; Herder and Schliewen 2010; von Rintelen et al. 2010). Furthermore, gene flow has been found to occur between lake and stream telmatherinid fish species and may play an important role in adaptive divergence (Herder et al. 2006; Schwarzer et al. 2008). While geographic proximity and hydrological connectivity of this chain of lakes allows dispersal among the interconnected basins, many species remain endemic to a single lake, suggesting that strong natural selection and local adaptation govern species distributions in the system (Vaillant et al. 2011). Such selection could be the result of environmental differences between the lakes. While dispersal of benthic organisms and fish among lakes is restricted to the river connections, planktonic species are known to disperse readily over local geographic scales (approximately 10 km) through a variety of mechanisms (Havel and Shurin 2004), eliminating geographic barriers as a determinant of species distributions.

In this study, we investigate patterns of speciation in the diaptomid (Copepoda: Calanoida) populations of Sulawesi using cytogenetic, phylogenetic, and population genetic analyses at the island, local (i.e., among the interconnected Malili lakes), and intralacustrine scales. We contrast the drivers of speciation in the relatively homogenous planktonic environment with the speciation forces observed in the more heterogeneous benthic and littoral habitats. We hypothesize that at the island scale, each isolated lake system has been colonized independently. Thus, we predict that each major basin will harbor a different, possibly endemic, species. In contrast to littoral and benthic organisms, we hypothesize that planktonic organisms will have little genetic differentiation at local and intralacustrine scale due to homogenous pelagic environments that allow unrestricted gene flow. Therefore, we predict panmictic intralacustrine populations as well as genetically homogenous populations among the interconnected Malili lakes.

## Materials and Methods

### Sample collection and genome size estimation

We surveyed five lakes on the island of Sulawesi, Indonesia: Lakes Tondano, Poso, and the three major lakes of the Malili lake system (Matano, Mahalona, and Towuti). Three to five sites were sampled per lake for a total of 19 sites (Table S1; Fig. 1A). Although copepods may be present up to 100 m depth in Lake Matano (Sabo et al. 2008), zooplankton samples were collected from the surface layer by 10 m vertical tows using a 62-μm-mesh plankton net (1 m diameter) and immediately preserved in 95% ethanol. The key of Reddy (1994) was used for taxonomic identification. As polyteny has been found as a potential source of cryptic speciation in copepods (McLaren et al. 1966), we estimated the nuclear DNA content (genome size) from each population using the Feulgen image analysis densitometry method described by Hardie et al. (2002). Genome size was estimated for four individuals from each lake population using a minimum of 20 and maximum of 50 nuclei. Optical densities were converted into picograms using chicken (*Gallus gallus domesticus*) blood as a standard. Means and standard errors were calculated from four individuals of each lake population. A one-way ANOVA and Tukey post hoc comparisons were performed with the STATISTICA v. 8.0 software package to test for differences between all lake populations.

### DNA extraction, amplification, and sequencing

Total genomic DNA (gDNA) was extracted from single individuals (adults or late copepodite stages) using a modified proteinase *K* method (Schwenk et al. 1998). A total of 417 individuals (accession nos: JX868096–JX868508 and JN183939–JN183943) were analyzed for the mitochondrial cytochrome *c* oxidase subunit I (COI) gene. A subset of these individuals, chosen to represent each of the major COI clades, were analyzed for the nuclear internal transcribed spacer 1 (ITS1) region (*N* = 49; accession nos: JX868047–JX868095), and 18S and 28S ribosomal RNA genes (*N* = 21; accession nos: JX868003–JX868046). All 20 μL polymerase chain reactions contained 1× PCR buffer (Genscript), 1 mmol/L MgCl_2_, 1.0 μmol/L of each primer, 0.08 μmol/L dNTPs, 0.4 units of Taq DNA polymerase (Genscript), and 100 ng gDNA. Primers and reaction temperature profiles are given in Table S4. Following amplification, PCR products were purified using the solid-phase reversible immobilization method (DeAngelis et al. 1995). Sequencing reactions were performed using forward primers and BigDye Terminator 3.1 chemistry on an ABI 3130XL automated sequencer (Applied Biosystems, Foster City, CA). Reverse primers were used to resolve ambiguous sequences.

### Phylogenetic and demographic analyses

Sequences for each marker were aligned and quality controlled using CodonCode Aligner v.2.0.6 (CodonCode Corporation, Dedham, MA). Neighbor-joining (NJ) and Bayesian (BI) phylogenetic reconstructions were conducted for three data sets: COI, ITS1, and concatenated 18S + 28S. NJ phylogenetic reconstructions were performed in MEGA v. 4 (Tamura et al. 2007) using the TrN substitution model and 10^3^ bootstrap replicates. BI reconstructions were conducted with MrBayes v.3.1 (Ronquist and Huelsenbeck 2003) using the best fit substitution models as determined by Modeltest v.3.7 (Posada and Krandall 1998), and consisted of four replicate runs with four chains of 10^7^ generations, discarding the first 25% as burn-in. The calanoid copepod *Leptodiaptomus siciloides*, collected from Lake Erie, Ontario, Canada, was used to root all trees.

We explored gene flow between the three Malili lakes populations (Matano, Mahalona, and Towuti) with coalescent simulations using the full COI alignment and the longest nonrecombining stretch of DNA from the ITS1 alignment in the program IMa2 (Hey 2010). We conducted five final runs of 10^6^ generations using priors 5× larger than those estimated via the user guidelines. Log-likelihood ratio tests were performed to infer migration between lakes.

Relationships among the COI haplotypes were further examined by constructing a statistical parsimony haplotype network at the 95% connection limit in TCS v.1.21 (Clement et al. 2000). The number of haplotypes (*N*_h_), haplotype diversity (*h*), nucleotide diversity (*π*), Tajima's *D*, and Fu's *F*_S_ was calculated for the COI data with DnaSP v.5 (Librado and Rozas 2009). Tajima's *D* statistic (Tajima 1989) was used to test evolution under neutrality or demographic changes for each of the major COI clades. Significantly negative *D* values indicate strong selection or a population bottleneck, whereas positive *D* values indicate balancing selection (Tajima 1989). Population demographic changes were further investigated using Fu's *F*_S_ (Fu 1997) and pairwise mismatch distributions (Rogers and Harpending 1992) computed with 10^4^ permutations in Arlequin v. 3.5 (Excoffier and Lischer 2010). Statistically significant negative *F*_S_ values and unimodal mismatch distributions indicate an excess of recent mutations and population expansion events (Fu 1997). Positive *F*_S_ values indicate lack of alleles or overdominant selection and multimodal distributions indicate demographic equilibrium. Further details regarding phylogenetic and coalescent analyses can be found in Appendix S1.

## Results

### Morphological identification and genome size analysis

Three morphological species of diaptomids were identified across the island, each belonging to a different genus and inhabiting a different lake system (Lake Tondano: *Phyllodiaptomus* sp. [V. Alekseev, pers. comm.]; Lake Poso: *Neodiaptomus lymphatus* [Brehm, 1933]; lakes Matano and Mahalona: *Eodiaptomus wolterecki matanensis* [Brehm, 1933]; and Lake Towuti: *Eodiaptomus wolterecki wolterecki* [Brehm, 1933]; Table S1). Our genome size estimates were constant (*P* > 0.97) among the Malili lakes populations eliminating polyteny as a potential source of cryptic speciation (Table S1).

### Sequence polymorphism and phylogenetic analyses

The COI alignment consisted of 511 base pairs (bp) and 418 sequences and contained 175 synonymous and four nonsynonymous mutations comprising 150 unique haplotypes. The mitochondrial phylogeny was used to select 49 representative individuals for sequencing the nuclear genes. The ITS1 alignment consisted of 708 bp and 49 sequences and contained 154 variable sites and 10 unique haplotypes, whereas the 980 bp 18S (352 bp) and 28S (628 bp) alignment consisted of 21 sequences with 36 variable sites and four unique haplotypes.

Phylogenetic tree topologies were consistent between NJ and BI inference methods. For each marker, BI analyses converged and produced low average standard deviation of split frequencies. The mitochondrial COI phylogeny reveals four well supported and highly divergent clades corresponding to populations in lakes Tondano (TD1, TD2), Poso (PO), Matano/Mahalona (MA/MH), and Towuti (TO) (Fig. 1B). Within Lake Tondano, there are two highly divergent lineages (15.4% sequence divergence; Table 1). The population of Lake Poso constitutes a single monophyletic group. The Malili lakes contain two divergent clades, MA/MH and TO. Individuals belonging to the MA/MH clade are found in lakes Matano and Mahalona, with four haplotypes shared between lakes. Lake Towuti harbors its own divergent lineage (12.7% sequence divergence from MA/MH; Table 1). Three individuals grouping with MA/MH were recovered at site TO1, where the River Tominanga flows into Lake Towuti, confirming that dispersal occurs between the lakes. There was no evidence for intralacustrine population structure as individuals from different sampling locations were evenly distributed within each clade.

**Table 1:** Mean Tamura–Nei sequence divergences between the major cytochrome *c* oxidase subunit I clades

	TD1	TD2	PO	MA/MH	TO
TD1	*0*				
TD2	0.154	*0.003*			
PO	0.205	0.204	*0.012*		
MA/MH	0.259	0.220	0.237	*0.017*	
TO	0.268	0.233	0.222	0.127	*0.011*

TD1, TD2, Tondano; PO, Poso; MA/MH, Matano/Mahalona; TO, Towuti.

Italic values along the diagonal are average within-group distances.

The nuclear phylogenies corroborate the divergences between genera, albeit with fewer informative sites because of highly conserved ribosomal sequences (Fig. 1C). However, there were two major discordances between the mitochondrial and nuclear trees. First, the two divergent mitochondrial clades in Lake Tondano (TD1, TD2) collapse into a single nuclear genotype. Second, both mitochondrial clades from the Malili lakes (MA/MH and TO) form a single nuclear clade with identical 18S and 28S sequences, and minimal variation in ITS1 sequences (0–0.6%).

Coalescent simulations in IMa2 (Hey 2010) provided evidence for gene flow among the Malili lakes populations. Over several pilot runs the posterior density distributions did not flatten and reach zero within prior bounds for all estimated parameters, indicating that the results are dependent on priors and should be interpreted with caution. In five final independent runs, migration rates converged and were consistently greater than zero for bidirectional migration between Matano and Mahalona and unidirectional migration from Mahalona to Towuti (Table S2). Log-likelihood ratio tests confirmed the rejection of a model with zero gene flow from Mahalona to Towuti (*P* = 0.016), indicating unidirectional gene flow from Lake Mahalona into Lake Towuti.

### Demographic and population genetic analyses

**Figure 2:**
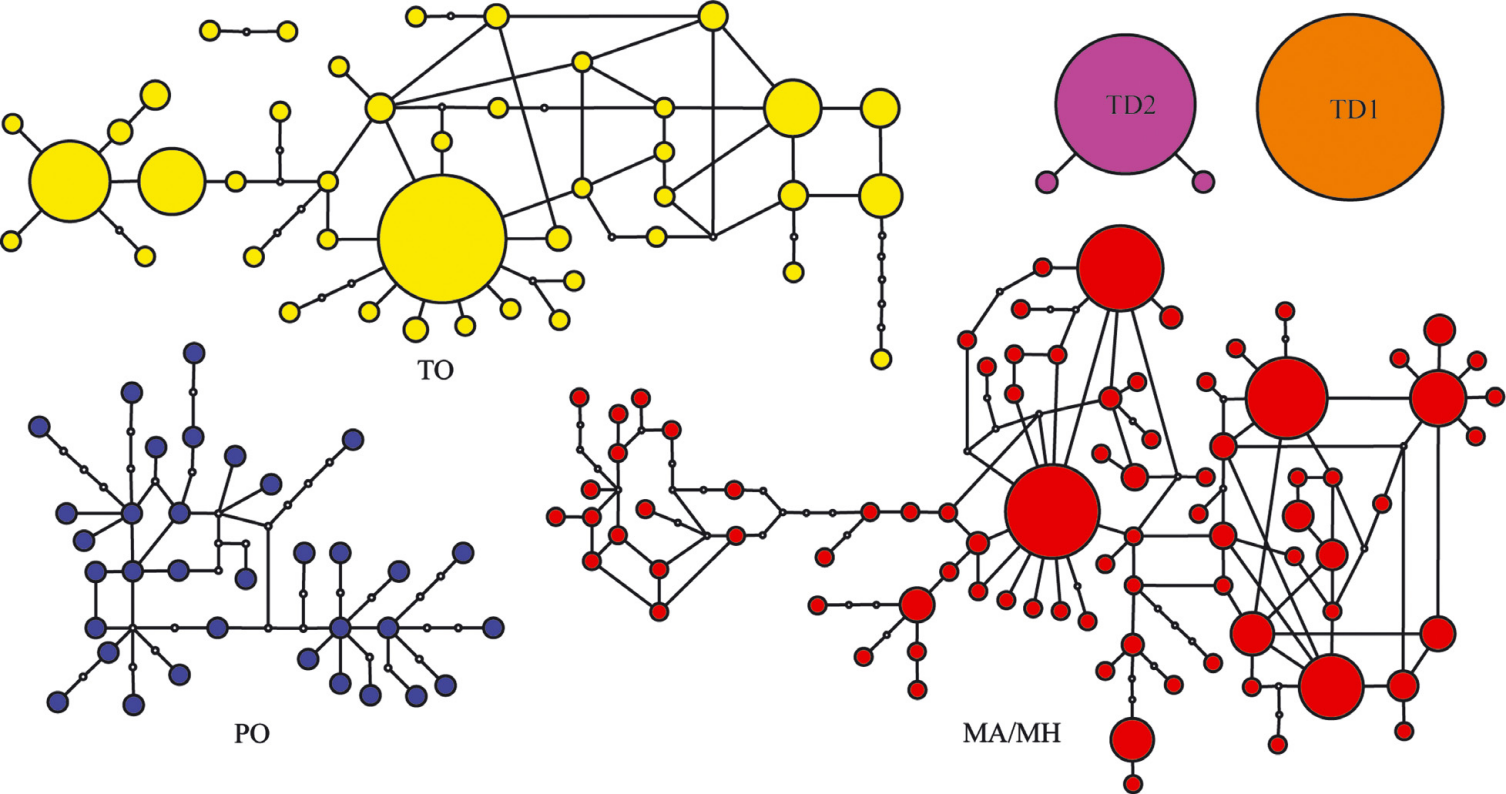
Maximum parsimony haplotype network for the cytochrome *c* oxidase subunit I (COI) gene. Colored circles represent haplotypes, with size corresponding to haplotype frequency. Single lines correspond to single mutation steps (i.e., 1 base pair change) and small open circles represent extinct or unsampled haplotypes.

The population of Lake Tondano was genetically impoverished with only two major haplotypes (*h* = 0.53), indicative of a very recent and severe bottleneck or selective sweep. Populations in Poso and the Malili lakes had very high haplotype diversities (*h* = 0.91–1.0), suggesting high genetic diversity and large effective population sizes. Most notably, Lake Poso had a haplotype diversity of 1.0, where every sampled individual possessed a unique haplotype. Tajima's *D* (Tajima 1989) was used to test for selection on the mitochondrial genome. Values were all negative but nonsignificant within all the major COI clades, indicating that the mitochondria is under purifying selection but otherwise evolving neutrally (Table S3). Populations from Poso, Matano/Mahalona, and Towuti had significantly negative *F*_S_ values indicating an excess of rare haplotypes (Fu 1997; Table S3). This was corroborated by the normal mismatch distributions and complex web-like (looping) structures of the maximum parsimony networks of each clade (Figs. 2, S1). In the MA/MH and TO clades there were several frequent haplotypes with many radiating branches, suggesting recent population expansion. There was no evidence for intralacustrine population structure as individuals from different sampling locations were evenly distributed within each network.

## Discussion

The large lakes of Sulawesi offer replicate systems to study speciation processes of zooplankton across various geographical and ecological settings. As predicted, each isolated lake system (Tondano, Poso, and Malili lakes) was colonized independently by a different genus of copepod, and each species is endemic to Sulawesi Island. Nearly all their congeners are inhabitants of Southeast Asia (Lai and Fernando 1980; Reddy 1994), indicating that long-term isolation and endemism dominate Sulawesi's zooplankton communities. The presence of a single diaptomid morphospecies in each of the three long-lived lake systems indicates that the sequence of colonization events is an important determinant of species distributions. Our findings are consistent with Dumont's (1994) postulation that in long-lived, tropical, oligotrophic lakes, a single generalist calanoid monopolizes resources by eventually outcompeting other pelagic grazers (e.g., cladocerans) and evolving to endemic status.

The diaptomids in Lake Poso support the prediction of a large, stable, panmictic population at the intralacustrine scale. The high haplotype diversity (*h* = 1) and sprawling haplotype network (Fig. 2) of this population indicate that genetic diversity and effective population size are extremely large. Thus, the significantly negative *F*_S_ value and unimodal mismatch distribution, indicative of population growth, are more likely the result of insufficient sampling of this massive, genetically diverse population. Furthermore, there was no evidence for intralacustrine population structure across sampling locations separated by more than 10 km. The nonsignificant Tajima's *D* (Table S3) and vast majority of synonymous mutations (98%) suggest purifying selection, but otherwise neutral evolution. All together, our results are a testament to the long-term stability of the Lake Poso diaptomid population. Compared to more diverse littoral fauna including pachychilid, rissooidean, and hydrobioid gastropods, as well as atyid shrimps for which radiations have been documented (von Rintelen et al. 2004, 2007; Haase and Bouchet 2006; Zielske et al. 2011), our results support the hypothesis that niche diversification is unlikely even in homogenous, seasonally stable, long-lived habitats.

The diaptomid population of Lake Tondano, however, revealed a very impoverished genetic diversity suggestive of a recent population bottleneck. We identified only two dominant haplotypes (*h* = 0.51) in this population. Such pronounced genetic scarcity may be the result of either very recent colonization, drastic changes in environmental conditions (e.g., volcanic eruptions or rapid lake-level fluctuations within the last 33,000 years; Dam et al. 2001), or exceptionally strong selection. The two dominant haplotypes identified were highly divergent (COI: 15.4%; Fig. 1B), fully sympatric, and nearly equal in abundance. Surprisingly, individuals carrying either dominant mitochondrial haplotype were identical at nuclear ribosomal loci. This indicates either unusually high heterogeneity in evolutionary rates of mitochondrial and nuclear genomes or relatively recent and extensive gene flow across the nuclear genome which has homogenized the nuclear ribosomal genes through concerted evolution (Hillis and Dixon 1991). Thus, Lake Tondano might be the site of very recent secondary contact and hybridization between genetically differentiated populations where competitive exclusion has been delayed by recombination and gene flow between lineages. Historically, smaller water bodies along the northern peninsula of Sulawesi may have harbored satellite populations and facilitated genetic divergence. This unusual observation provides a unique opportunity to investigate selection and drift on two coexisting mitochondrial lineages in a natural population that is geographically well defined and inhabits a relatively homogeneous environment.

The most interesting case of population structure is observed in the ancient Malili lakes where two divergent mitochondrial clades are geographically restricted to specific lakes. Considering each lake individually, high haplotype diversities (*h* = 0.91–0.95) and sprawling haplotype networks (Fig. 2) indicate extremely large, genetically diverse populations similar to Lake Poso. Likewise, the lack of genetic differentiation between sampling locations suggests high dispersal and panmixia within lakes. Significantly negative *F*_S_ values and unimodal mismatch distributions indicate recent population growth, but may again stem from insufficient sampling of these very large and extremely diverse populations. Therefore, the population of each individual lake supports our hypothesis of constrained intralacustrine genetic differentiation.

Among the Malili lakes, two highly divergent mitochondrial clades (COI: 12.7%; Fig. 1B) showed strong phylogeographic structure. The MA/MH clade was found only in the upstream Lakes Matano and Mahalona, whereas the TO clade was confined to the downstream Lake Towuti. This finding is contrary to dispersal theory, as short geographic distance and water flow between the lakes (<10 km), and the high dispersal ability of zooplankton allows for continuous dispersal of propagules between lakes. Such high dispersal potential should result in community homogenization as has been found in other studies of zooplankton (Havel and Shurin 2004). Several individuals belonging to the MA/MH clade were observed at the mouth of the Tominanga River in Lake Towuti, indicating dispersal does occur, yet mitochondrial haplotypes from upstream populations are unable to penetrate the downstream habitat. Furthermore, there was little variation in the ITS1 region and no divergence in the nuclear ribosomal genes between populations of the three Malili Lakes (Fig. 1C). Our coalescent analyses found significant gene flow between the MA/MH and TO clades, indicating that the homogeneity of nuclear loci is the result of recent or ongoing gene flow, rather than an exceptionally slow evolutionary rate for the nuclear loci (i.e., incomplete lineage sorting). Moreover, this gene flow was unidirectional from MA/MH into TO, consistent with the north to south direction of water flow through the system (i.e., from Mahalona into Towuti), suggesting that the rivers are the important vectors of dispersal. Our results indicate that the Malili diaptomids maintain distinct populations despite potential for continuous dispersal and gene flow.

This phylogeographic pattern raises two major evolutionary questions. First, how did the divergence between mitochondrial clades arise? And second, if gene flow occurs across the nuclear genome, which forces maintain the geographic separation of the two mitochondrial clades? Several evolutionary scenarios could create the observed pattern. First, the strong north–south phylogeographic structure could result from the dispersal of males only. However, this scenario is very unlikely as adults of both sexes were found in the Tominanga River that connects the two divergent clades and at the downstream mouth of the river in Lake Towuti. Another possibility is that a recent selective sweep or colonization event has rapidly replaced the mitochondrial genome of one population (Hurst and Jiggins 2005). However, such drastic demographic events would likely produce notable genetic bottlenecks. This is inconsistent with the very high haplotype diversities, low Tajima's *D* values, negative Fu's *F*_S_, and webbing parsimony networks which indicate that both clades represent large, stable, neutrally evolving populations.

A more likely evolutionary scenario is that the formation of the two clades in the Malili lakes involves strong natural selection due to different selection regimes of lake-specific environments. Natural selection is implicated as a driver of adaptive radiation in the other species flocks of the Malili lakes. The radiations of the shrimp, crab, gastropod, and fish species are all characterized by trophic specialization (Glaubrecht and von Rintelen 2008; Schubart and Ng 2008; Herder and Schliewen 2010; von Rintelen et al. 2010). Many of these species show high genetic differentiation among the Malili lakes and remain endemic to a single lake, further indicating that local adaptation plays vital role in species distributions (Vaillant et al. 2011; von Rintelen et al. 2011). In Lake Matano, stable isotope analyses indicated that the diaptomids use alternate food sources such as microbes and detritus concentrated at the 100 m chemocline in addition to phytoplankton (Sabo 2006). As Towuti lacks stratification and harbors a more abundant forage fish community (Parenti and Soeroto 2004; Walter et al. 2011), both resource availability and predation pressures differ among the lakes. Thus, strong natural selection resulting from both physicochemical and ecological differences among lakes might have promoted adaptation and divergence between the two diaptomid mitochondrial clades. Historical periods of geographic isolation between lakes may have also contributed to reduced gene flow and facilitated genetic divergence of Malili species flocks, particularly in benthic lineages with poor dispersal ability. However, copepods exhibit very high passive dispersal ability that generates significant gene flow across small geographic ranges of tens of kilometers (Boileau and Hebert 1991). Thus, it is unlikely that geographic isolation had a dominant role in shaping the observed genetic structure of the Malili populations.

**Figure 3:**
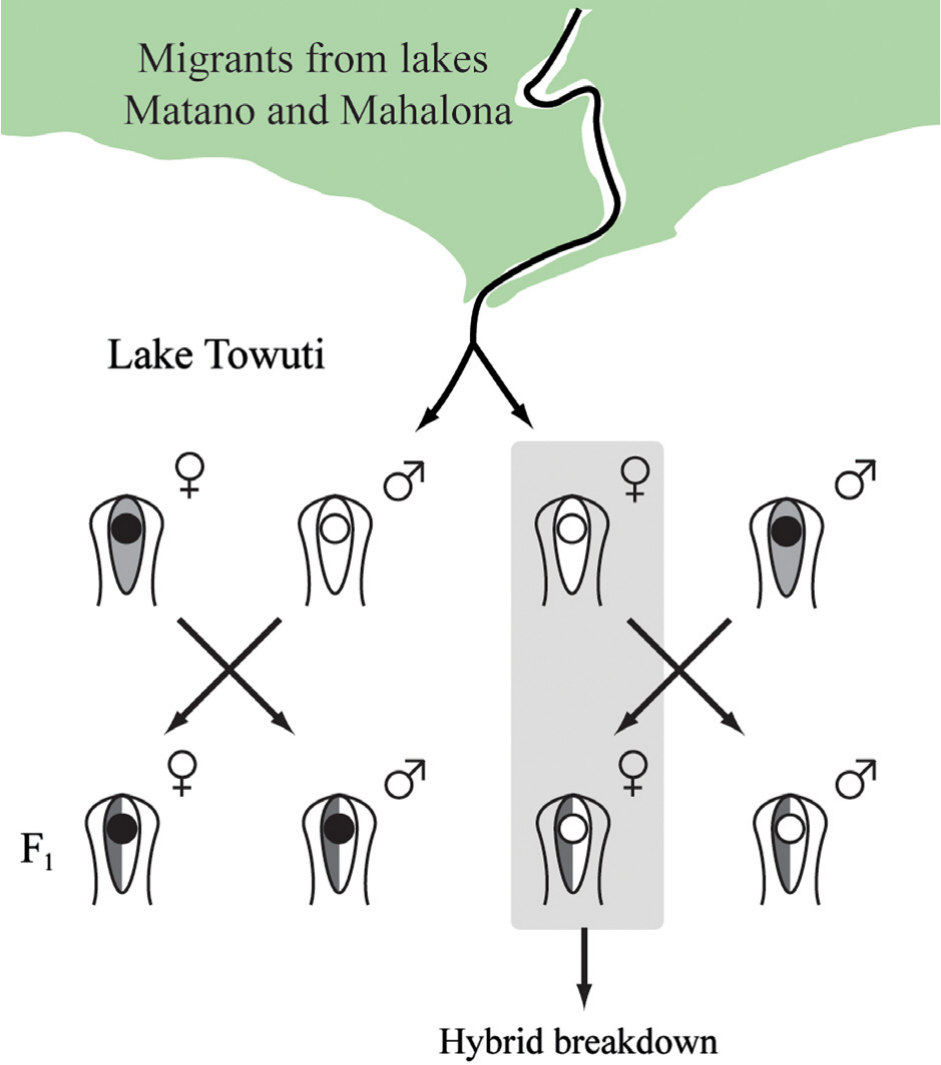
Proposed model for restricted gene flow between populations of the two Malili clades. White and gray bodies represent MA/MH and TO nuclear genomes, respectively, and white and black circles represent MA/MH and TO mitochondria, respectively. The maternal lineage of migrants (gray box) will suffer hybrid breakdown as their mitochondria find themselves in an ever-increasing divergent nuclear background after repeated backcrossing. All other hybrid crosses will regain native mitochondria and progressively disseminate migrant genes into the population with each backcross.

Contemporary phylogeographic separation of the clades would be maintained by strong selection against migrant and hybrid genotypes. In addition to natural selection against hybrids due to differences in lake habitat, reproductive barriers could arise from mitonuclear incompatibilities or other cytoplasmically inherited elements (Ballard and Whitlock 2004; Hurst and Jiggins 2005; Gershoni et al. 2009; Burton and Barreto 2012). Dysfunctional interactions between maternally inherited mitochondria and the nuclear genes of a divergent lineage have been demonstrated as the cause of hybrid breakdown between divergent populations of the harpacticoid copepod *Tigriopus californicus* (Ellison and Burton 2008). This mechanism could further maintain the strong north/south segregation of the Malili mitochondrial clades through asymmetrical hybrid breakdown in the maternal lineage. Under this scenario, the female progeny of migrant females (i.e., the maternal lineage) inherits migrant mitochondria and suffers breakdown through repeated backcrossing with a divergent population, whereas the hybrid progeny of migrant males (which inherit local mitochondria) acts as vehicles for nuclear gene flow between the populations (Fig. 3). The significant gene flow between the MA/MH and TO clades detected by our coalescent analysis supports this hypothesis and indicates that the homogeneity of nuclear loci is the result of gene flow, rather than incomplete lineage sorting. Moreover, the unidirectional gene flow from MA/MH into TO, consistent with the north to south direction of water flow through the system provides further support to our proposed model for restricted gene flow between populations of the two Malili clades (Fig. 3).

Overall, our study suggests that colonization order, local adaptation, and priority effects are critical for determining the distributions of freshwater zooplankton at large scales. The copepod populations in Lake Poso and the Malili lakes demonstrate that intralacustrine speciation in a homogenous environment is unlikely even in long-lived habitats, in agreement with previous observations of lack of radiation in pelagic calanoids of other ancient lakes (Dumont 1994). However, the populations of Lake Tondano suggests that stochastic events like local extinctions, colonizations, and bottlenecks may play a critical role in planktonic speciation by altering population genetic structure through intense drift and selection as well as facilitating hybridization between divergent lineages.

While pelagic freshwater copepods have been found to exhibit large mitochondrial sequence divergences (up to 15–25%) over regional geographic scales (Adamowicz et al. 2007; Makino and Tanabe 2009; Thum and Harrison 2009; Makino et al. 2010; Marrone et al. 2010), the Malili diaptomids show that high genetic divergence (12.7%) can form and be maintained over extremely small geographic scales (i.e., <10 km) despite high potential for dispersal and gene flow. It has been suggested that low rates of gene flow between partially isolated and genetically differentiated populations may accelerate speciation or adaptation by providing an influx of novel genotypes and generating greater phenotypic diversity (Seehausen 2004; Mallet 2007). Because freshwater plankton often occupy neighboring, insular habitats with specific ecology and rich deposits of resting eggs, intermittent dispersal between populations, low levels of gene flow, and natural selection may act in concert to drive adaptation in this group. Although the forces responsible for differentiation in the Malili diaptomids are complex, the populations are clearly in the process of speciation. Species divergence proceeds despite the high level of dispersal and gene flow between the interconnected habitats. The Malili lakes represent an isolated and replicated system with the potential to reveal much about the role of local adaptation and hybridization between differentiated populations.

## Data Accessibility

All DNA sequences used in this study are archived in GenBank.

COI: JX868096–JX868508 and JN183939–JN183943.

ITS1: JX868047–JX868095.

18S+28S: JX868003–JX868046.

Data matrices and phylogenetic trees are available on TreeBASE.

Study ID: 14421.

## Supporting Information

**Figure S1.** Observed (open circles) and expected (closed circles) pairwise mismatch distributions of COI sequences of diaptomid populations from lakes Tondano, Poso, and the Malili lakes.

**Appendix S1.** Materials and methods.

**Table S1.** Mitochondrial DNA genetic diversity indices and DNA content (2C) of diaptomid populations from Sulawesi.

**Table S2.** Migration rate parameter estimates (marginal peak locations) for five independent runs of IMa2 and the results of log-likelihood ratio tests against a model with no migration.

**Table S3.** Results of population demographic analyses for Tajima's *D*, Fu's *F*_S_, and mismatch distributions.

**Table S4.** Primer pairs and thermal cycling programs used for the COI, ITS1, 18S, and 28S amplification.
