# Supplementary material for: Speciation patterns and processes in the zooplankton of the ancient lakes of Sulawesi Island, Indonesia
Source: Ecol Evol. 2013 Aug 1;3(9):3083–94. doi: 10.1002/ece3.697 (PMC3790553; doi:10.1002/ece3.697)
Supplement: Supplementary file 1 [file ece30003-3083-SD1.doc]

**SI Appendix**

**Materials and Methods**

*Phylogenetic and coalescent analyses*

The 18S and 28S alignments were concatenated following a non-significant incongruence length difference test (ILD p = 1.0; Farris *et al.* 1995), with 1000 homogeneity replicates and a heuristic search algorithm (with default settings) in PAUP*v. 4.0b.10 (Swofford 2002). Polymorphic sites and mutation biases were analyzed with DnaSP v. 5 (Librado and Rozas 2009). The best model of evolution for each dataset (COI: TrN+I+G; ITS1: TVM+G; 28S+18S: TrN), was determined using Modeltest v. 3.7 (Posada and Krandall 1998) under the Akaike Information Criterion (Posada and Buckley 2004). For coalescent simulations in IMa2, pilot runs of 100,000 generations were conducted with priors for population sizes, divergence times, and migration rates chosen as per the user guidelines, as well as priors 5x, 10x, and 15x larger. Small parameter autocorrelations, trendplots, and large ESS estimates indicated good chain mixing. In all pilot runs, the posterior probability distributions for some of the parameters varied and did not flatten to zero, indicating dependence on priors and low reliability of parameter estimates. However, the shapes of the probability distributions were similar between runs, indicating consistent general patterns in the data (i.e. non-zero migration rates). We used the following command lines:

*M mode:* ima2.exe -i datafull.txt -b 300000 -d 10 -hfg -hn 80 -ha 0.9 -hb 0.8 -l 100000 -m 4.5 -o datafulloutput.txt -p 2567 -r 25 -t 22 -q 54.5 -z 100

*L mode:* ima2.exe -c 12 -i datafull.txt -b 300000 -l 100000 -m 4.5 -o lmodedatafull.txt -p 35 -r 0 -t 22 -q 54.5 -u 0.05 -w nestedmodels.txt -v test1datafull

**Supplemental References**

Crease TJ, Colbourne JK (1998) The unusually long small-subunit ribosomal RNA of the Crustacean, *Daphnia pulex*: sequence and predicted secondary structure. *Journal of Molecular Evolution*, **46**, 307–313.

Farris JS, Kallersjo M, Kluge AG, Bult C (1995) Constructing a Significance Test for Incongruence. *Systematic Biology*, **44**, 570–572.

Folmer O, Black M, Hoeh W, Lutz R, Vrijenhoek R (1994) DNA primers for amplification of mitochondrial cytochrome c oxidase subunit I from diverse metazoan invertebrates. *Molecular Marine Biology and Biotechnology*, **3**, 294–299.

Hillis DM, Dixon MT (1991) Ribosomal DNA: molecular evolution and phylogenetic inference. *Quarterly Review of Biology*, **66**, 411–453.

Librado P, Rozas J (2009) DnaSP v5: a software for comprehensive analysis of DNA polymorphism data. *Bioinformatics*, **25**, 1451–1452.

Omillian AR, Taylor DJ (2001) Rate Acceleration and Long-branch Attraction in a Conserved Gene of Cryptic Daphniid (Crustacea) Species. *Molecular Biology and Evolution*, **18**, 2201–2212.

Posada D, Buckley TR (2004) Model Selection and Model Averaging in Phylogenetics: Advantages of Akaike Information Criterion and Bayesian Approaches Over Likelihood Ratio Tests. *Systematic Biology*, **53**, 793–808.

Posada D, Krandall KA (1998) MODELTEST: testing the model of DNA substitution. *Bioinformatics*, **14**, 817–818.

Swofford DL (2002) PAUP*: Phylogenetic Analysis Using Parsimony (* and Other Methods). Version 4.0b.10. Sinauer Associates Inc, Sunderland, MA.

White TJ, Bruns T, Lee S, Taylor J (1990) Amplification and direct sequencing of fungal ribosomal RNA genes for phylogenetics. In: *PCR Protocols: A Guide to Methods and Applications* (eds. M. Innis, D. Gelfand, J. Sninsky, T. White), pp. 315–322. Academic Press, San Diego, California.

**Figure Captions**

**Figure S1** Observed (open circles) and expected (closed circles) pairwise mismatch distributions of COI sequences of diaptomid populations from lakes Tondano, Poso, and the Malili lakes. Unimodal distributions indicate population growth.

**Figure S1**


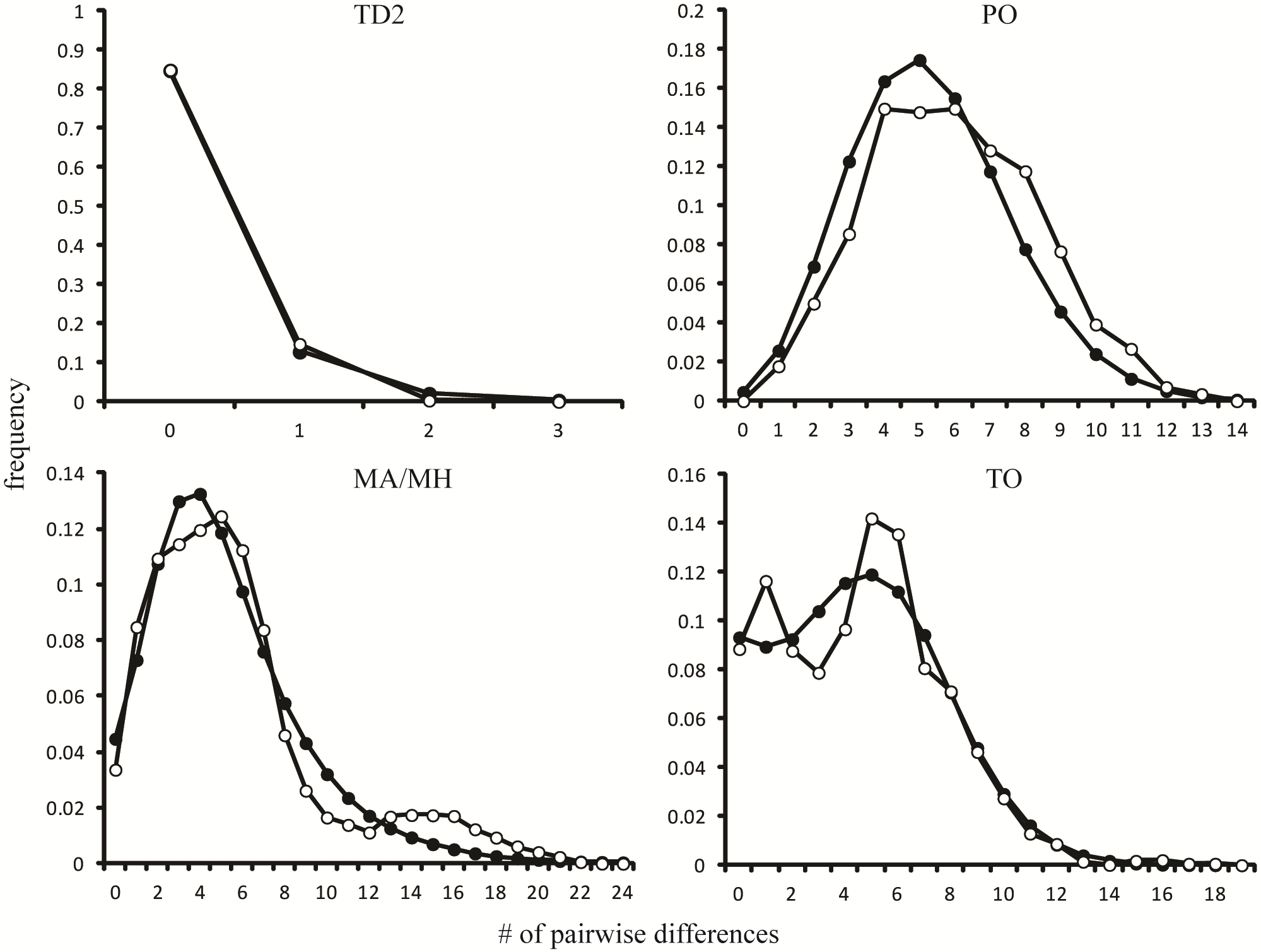


**Table S1** Mitochondrial DNA genetic diversity indices and DNA content (2C) of diaptomid populations from Sulawesi. *N*, number of individuals; *N*_h_, number of haplotypes; *h*, haplotype diversity; *π*, nucleotide diversity.

| **Species name /**  **sampling site** | **ID** | **Latitude** | **Longitude** | ***N*** | ***N*_h_** | ***h*** | ***π*** | **Mean DNA content (pg ± SE)** |
| --- | --- | --- | --- | --- | --- | --- | --- | --- |
| *Phyllodiaptomus* sp. | **TD** |  |  | **59** | **4** | **0.530** | **0.068** | 0.81 ± 0.02 |
| Tondano East | TD1 | 1°13'02.57"N | 124°54'05.59"E | 24 | 2 | 0.507 | 0.068 |  |
| Tondano Center | TD2 | 1°13'09.03"N | 124°53'39.65"E | 20 | 4 | 0.574 | 0.068 |  |
| Tondano West | TD3 | 1°13'09.11"N | 124°53'06.57"E | 15 | 2 | 0.533 | 0.072 |  |
| *Neodiaptomus lymphatus* | **PO** |  |  | **34** | **34** | **1.0** | **0.012** | 0.67 ± 0.03 |
| Poso North | PO1 | 1°48'05.36"S | 120°37'00.38"E | 5 | 5 | 1.0 | 0.011 |  |
| Poso Center | PO2 | 1°54'09.69"S | 120°37'01.91"E | 22 | 22 | 1.0 | 0.013 |  |
| Poso South | PO3 | 2°00'07.51"S | 120°40'06.39"E | 7 | 7 | 1.0 | 0.008 |  |
| *Eodiaptomus wolterecki* *matanensis* | **MA** |  |  | **99** | **37** | **0.947** | **0.006** | 0.75 ± 0.01 |
| Matano Northwest | MA1 | 2°27'00.79"S | 121°15'00.33"E | 26 | 15 | 0.945 | 0.005 |  |
| Matano Center | MA2 | 2°28'01.18"S | 121°18'00.93"E | 42 | 19 | 0.906 | 0.005 |  |
| Matano Southeast | MA3 | 2°30'02.66"S | 121°24'09.99"E | 16 | 9 | 0.867 | 0.004 |  |
| Petea outlet | MA4 | 2°32'06.07"S | 121°28'08.50"E | 15 | 9 | 0.924 | 0.006 |  |
| *Eodiaptomus wolterecki matanensis* | **MH** |  |  | **96** | **50** | **0.936** | **0.011** | 0.74 ± 0.03 |
| River Petea | MH1 | 2°33'16.30"S | 121°31'23.92"E | 3 | 3 | 1.0 | 0.005 |  |
| Petea mouth | MH2 | 2°34'24.61"S | 121°30'29.96"E | 22 | 10 | 0.814 | 0.007 |  |
| Mahalona East | MH3 | 2°34'33.90"S | 121°30'16.23"E | 31 | 24 | 0.961 | 0.017 |  |
| Mahalona Center | MH4 | 2°35'15.60"S | 121°29'36.40"E | 25 | 14 | 0.893 | 0.008 |  |
| Mahalona West | MH5 | 2°35'43.74"S | 121°28'52.06"E | 15 | 9 | 0.905 | 0.008 |  |
| *Eodiaptomus wolterecki wolterecki* | **TO** |  |  | **129** | **44** | **0.913** | **0.012** | 0.73 ± 0.02 |
| Tominanga mouth | TO1 | 2°39'58.10"S | 121°31'32.51"E | 15 | 11 | 0.908 | 0.031 |  |
| Towuti North | TO2 | 2°42'05.83"S | 121°35'02.41"E | 17 | 12 | 0.941 | 0.007 |  |
| Towuti East | TO3 | 2°48'08.43"S | 121°33'01.92"E | 13 | 9 | 0.910 | 0.007 |  |
| Towuti West | TO4 | 2°49'08.95"S | 121°27'06.55"E | 84 | 27 | 0.902 | 0.009 |  |
| **Total** |  |  |  | **417** | **153** | **0.971** | **0.107** |  |

**Table S2** Migration rate parameter estimates (marginal peak locations) for 5 independent runs of IMa2 and the results of log-likelihood ratio tests against a model with no migration. Migration estimates are in units of genes/generation. Asterisks denote significant log-likelihood ratio tests (i.e. where the model with migration fits the data significantly better than a model with no migration). The populations are labelled MA=Matano, MH=Mahalona, TO=Towuti.

| Parameter: | MH>MA | LLR | MA>MH | LLR | TO>MA | LLR | MA>TO | LLR | TO>MH | LLR | MH>TO | LLR |
| --- | --- | --- | --- | --- | --- | --- | --- | --- | --- | --- | --- | --- |
| Run 1 | 0.176 | 0.052 ns | 0.252 | **4.351*** | 0 | 0.000 ns | 0 | 0.000 ns | 0 | 0.000 ns | 0.246 | **4.880*** |
| Run 2 | 0.216 | 0.145 ns | 0.271 | **3.619*** | 0 | 0.000 ns | 0 | 0.000 ns | 0 | 0.000 ns | 0.251 | **4.487*** |
| Run 3 | 0.252 | 0.134 ns | 0.269 | **4.439*** | 0 | 0.000 ns | 0 | 0.000 ns | 0 | 0.000 ns | 0.251 | **3.952*** |
| Run 4 | 0.373 | 0.162 ns | 0.257 | **3.590*** | 0 | 0.000 ns | 0 | 0.000 ns | 0 | 0.000 ns | 0.25 | **3.941*** |
| Run 5 | 0.296 | 0.182 ns | 0.24 | **3.173*** | 0 | 0.000 ns | 0 | 0.000 ns | 0 | 0.000 ns | 0.246 | **4.134*** |

**Table S3** Results of population demographic analyses for Tajima’s *D*, Fu’s *F_S_*, and mismatch distributions. Significant values are highlighted in bold.

| Clade | Tajima’s *D* | | Fu’s *F_S_* | | Mismatch distribution | |
| --- | --- | --- | --- | --- | --- | --- |
|  | *D* | p | *F_S_* | p | τ | p |
| TD1 | n/a | - | n/a | - | n/a | - |
| TD2 | -1.513 | > 0.10 | -2.176 | 0.087 | 3.00 | 0.378 |
| PO | -1.710 | > 0.05 | **-34.447** | **< 0.001** | 5.324 | 0.169 |
| MA/MH | -1.551 | > 0.05 | **-86.316** | **< 0.001** | 2.916 | 0.616 |
| TO | -0.397 | > 0.10 | **-20.257** | **< 0.001** | 6.365 | 0.872 |

**Table S4** Primer pairs and thermal cycling programs used for the COI, ITS1, 18S, and 28S amplification. Locality names are defined in Table S1.

| **Marker** | **Population** | **Primer pair** | **Amplicon size (bp)** | **Sequence (5'-3')** | **Source** | **Cycling program** |
| --- | --- | --- | --- | --- | --- | --- |
| COI | TD, MA, TO | HCO2198 | 680 | taa act tca ggg tga cca aaa aat ca | Folmer *et al*. 1994 | 94° for 3m;  5 cycles of [94° for 45s, 45° for 45s, 72° for 45s];  30 cycles of [94° for 45s, 50° for 45s, 72° for 45s];  72° for 4m |
|  |  | LCO1490 |  | GGT CAA CAA ATC ATA AAG ATAT TGG |  |  |
|  | PO | HCO2198 | 625 | taa act tca ggg tga cca aaa aat ca | Folmer *et al.* 1994 |  |
|  |  | SWR1 |  | TAT TTA ATT GCW GGY GCT TG | This study |  |
|  | MH | SWF1a | 585 | TAT AGT AAT TGC KCC YGC T | This study |  |
|  |  | LCO1490 |  | GGT CAA CAA ATC ATA AAG ATAT TGG | Folmer *et al.* 1994 |  |
| ITS1 | All lakes | ITS5 | 765 | GGA AGT AAA AGT CGT AAC AAG G | White *et al.* 1990 | 95° for 1m;  35 cycles of [95°for 30s, 50° for 30s, 72° for 50s];  72° for 7m |
|  |  | ITS4 |  | TCC TCC GCT TAT TGA TAT GC |  |  |
| 18S | All lakes | SSU-531 | 385 | CCG AGG CCC CGT GAT TGG AAT GAG | Crease and Colbourne 1998 | 94° for 4m;  2 cycles of [94° for 30s, 60° for 45s, 72° for 3m];  5 cycles of [93° for 30s, 55° for 45s, 72° for 3m];  29 cycles of [93° for 30s, 50° for 1m, 72° for 3m];  72° for 8m |
|  |  | SSU-1085R |  | CTC TGT CGC CGC AGT ACG AAT GCC |  |  |
| 28S | All lakes | 28ee | 1280 | ATC CGC TAA GGA GTG TGT AAC AAC TCA CC | Hillis and Dixon 1991 | 40 cycles of [94° for 30s, 55° for 30s, 72° for 1.5m];  72° for 6m |
|  |  | D8r |  | GAG TCA AGC TCA ACA GGG TCT TCT TTC CC | Omilian and Taylor 2001 |  |
