# Supplementary figures and images for: Speciation patterns and processes in the zooplankton of the ancient lakes of Sulawesi Island, Indonesia
Source: Ecol Evol. 2013 Aug 1;3(9):3083–94. doi: 10.1002/ece3.697 (PMC3790553; doi:10.1002/ece3.697)

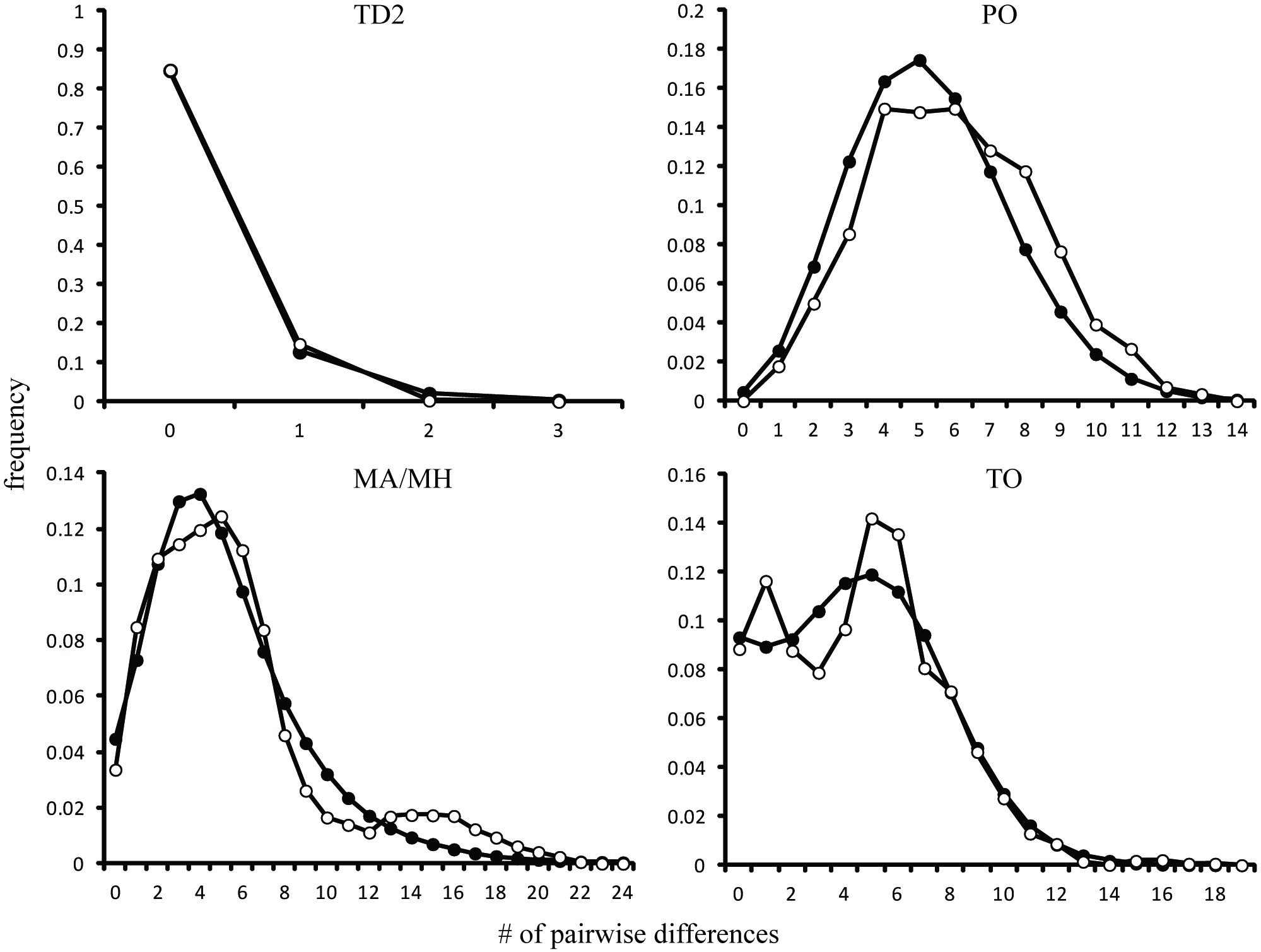

Supplement: Supplementary file 2 [file ece30003-3083-SD2.tif]
